# Autism Spectrum Disorder and Amplified Pain

**DOI:** 10.1155/2015/930874

**Published:** 2015-05-06

**Authors:** Ciaran Clarke

**Affiliations:** H.S.E. Dublin North City & County C.A.M.H.S., Metropolitan Building, James Joyce Street, Dublin 1, Ireland

## Abstract

Among the core features of ASD, altered sensitivities in all modalities have been accorded increasing importance. Heightened sensitivity to pain and unusual expressions of and reaction to pain have not hitherto been widely recognised as a presenting feature of ASD in general paediatrics. Failure to recognise ASD as a common cause of pain can lead to late diagnosis, inappropriate treatment, distress, and further disability. Two cases are presented which illustrate the late presentation of Autism Spectrum Disorder (Asperger's Syndrome subtype) with chronic unusual pain. *Conclusion*. Pain in autism can be atypical in its experience and expression and for this reason may go unrecognised by physicians treating chronic pain disorders.

## 1. Introduction

Autism Spectrum Disorder (ASD) is a neurodevelopmental disorder characterised by asynchronous development in several areas (e.g., language, communication, social relations, behavioural rigidity, motor abnormalities, cognitive capabilities, and sensory responsiveness). It is now recognised as a spectrum of disorders including Asperger's Syndrome [1]. With a prevalence of around 1% [2–4] ASD can no longer be considered rare. Among the core features of ASD, altered sensitivities in all modalities have been accorded increasing importance [1]. Among the sensory abnormalities recognised as important features of autism is either heightened or reduced sensitivity to pain. Individuals with ASD may experience pain in unusual ways, and their communication and social difficulties may make it difficult for them to make their distress known. This can lead to a delay in diagnosis when ASD present with heightened pain. We present two cases which highlight this problem.

## 2. Materials and Methods

Two cases of adolescents are presented with Autism Spectrum Disorder (Asperger's Disorder) who were diagnosed later, having presented earlier to rheumatologists.* Procedures followed *(particularly with regard to consent, data protection, and confidentiality) were in accordance with the ethical standards of the H.S.E. research ethics committee and with the Helsinki Declaration of 1975, as revised in 2000 (5).

## 3. Case  1

B.D. is a 15-year-old girl who presented to a paediatric rheumatology service with generalised aches and pains. Pain had been present for many years with fluctuating severity. She suffered from social anxiety, and the presence of others often made her pain worse. Oversensitivity to painful stimuli, or rather the experience of tactile stimuli such as traction and pressure as painful, had been a feature since infancy. She was particularly sensitive in the head and neck and was unable to tolerate even having her hair cut and styled. It was noteworthy that B.D. reported fluctuations in pain in response to stressful social situations.

She had also come to find almost every food unpleasant in taste and texture and, contemporaneous with times of greater pain, was able to tolerate only tasteless foods, such as dried noodles. Significant also was that this girl's diminished sensitivity to taste also varied with the degree of her distress and pain.

Despite an extensive diagnostic workup by a paediatric rheumatology service, no diagnosis was made. She was treated with nonsteroidal anti-inflammatory drugs, to little effect. “Amplified Pain Syndrome” was diagnosed. Physiotherapy was attempted but this exacerbated her limb pain and general discomfort and distress.

She presented to mental health services one year later because she was experiencing increasing antipathy towards family and peers of such intensity that she feared she might harm them. This fear was associated with increased generalised pain and discomfort and led her to refuse to attend school. Her somatic discomfort and pain remitted somewhat when she was away from school. She became sad and began to superficially cut her forearms.

She had always had difficulty in reciprocal social relations. During the assessment she said, “I just do not ‘get' people – they are just empty shells.” She had one genuine friend, whom she was content to see only every six months or so, and she was unable to describe the difference between a “friend” and an “acquaintance.”

She was outstanding throughout childhood for intelligence in music, art, mathematics, physics, and computer programming.

She had an overriding, absorbing interest with gross impairment in a very successful musical show. Her interest was so intense that she dressed as character in 18th century costume.

At assessment she wore the clothes described above. Her speech had reduced tonality. She rarely looked at persons she was addressing, and her shared gaze was very poor. She did not complain of pain during the neurodevelopmental assessment, though her mother afterwards reported that this was how she frequently experienced return to school. There was no self-injurious behaviour or unusual exploration of her environment, but increasing neck pain was reported towards the end of the long assessment, which she reported as being a stressful one.

Her mother was a language and linguistics scholar of exceptional ability, as evidenced by a distinguished publishing record in her chosen specialty. During the neurodevelopmental history she was noted to have a mild impairment in qualitative social interaction. In terms of family history of neurodevelopmental disorders, a maternal cousin had autism, and a brother had dyslexia.

A diagnosis of ASD (Asperger's Syndrome subtype) was made using the A.D.O.S. (Autism Diagnostic Observation Schedule) assessment tool and the S.C.Q. (Social Communications Questionnaire) and the S.R.S. (Social Responsiveness Scale) questionnaires.

## 4. Case  2

D.G. is a 16-year-old boy who presented to a paediatric rheumatology service at the age of 14 because of generalised muscle aches and joint pains. Post-viral reactive myalgia was diagnosed, though without serological evidence. There was no clinical or laboratory evidence of inflammatory disease. He had had a history of vague lower limb pain waxing and waning over several years. He was referred to mental health services some eighteen months later because of social withdrawal and school refusal. He had barricaded himself in his bedroom.

His mother reported that he had had unusual sensory responses at an early age. He was unable to tolerate meat that had not been pureed. He seemed to derive pleasure from the physical sensation of being squeezed. He had an inordinate preference for cold weather such that he would play outside in t-shirt and shorts even in near-zero temperatures. He developed secondary encopresis for approximately one year at the age of 5 and derived pleasure from faecal smearing. He was exquisitely sensitive to minor traction on his scalp such that his mother had to take special precautions when cutting his hair.

He had always had difficulties interacting with adults and with other children. He frequently thought other children were bullying him, when there was no evidence of this from several independent sources. His speech, though structurally normal, was pedantic and sophisticated (“like a barrister's,” his mother said). Although he had one or two genuine friends, he was happy with infrequent contact (every 3–6 months) with the one he liked most. He was naive and spoke about intimate things with excessive candour.

A diagnosis of ASD (Asperger's Syndrome) was made using the Royal College of Psychiatrists Diagnostic Interview for the Assessment of Adults with Autism Spectrum Disorder.

## 5. Discussion

Although both of these children had severe impairments in the domains of social relationships, pragmatic language, behavioural rigidity, and altered sensitivities, review of their medical records and parental interviews showed that an autistic disorder was not considered as a possible cause. This may be because of the atypical way in which autistic individuals sometimes experience and express pain, to altered sensory thresholds in some people with ASD, or to the relative unfamiliarity of many doctors and healthcare professionals with the wide variety of features with which autistic disorders can present.

Altered pain thresholds are a recognised feature of the sensory hypo- and hyperresponsiveness associated with Autism Spectrum Disorders (ASD). Although it is commonly assumed that reduced sensitivity to pain is more common [5], this may be because of other autism-related impairments [6]. Allely [7] has drawn attention to the unusual ways in which autistic individuals experience pain, for example, denying pain but describing such noxious stimuli as dental extraction as “discomfort.” Oversensitivity to pain has been shown to lead to a delay in diagnosis, as compared with autistic disorders in general [8]. For this reason, when enquiring about pain, it may be useful to be mindful of a wider vocabulary, enquiring about “discomfort,” “anxiety,” and so forth. The mechanism of altered pain sensitivities in autism (e.g., endogenous opioid excess, sensorial abnormalities, and sociocommunicative impairments) requires further elucidation [9].

Cross-species affective neuroscience studies confirm that primary-process emotional feelings are organized within primitive subcortical regions of the brain that are anatomically, neurochemically, and functionally homologous in all mammals that have been studied [10]. It may not be surprising, therefore, that in a neurodevelopmental disorder such as autism, in which problems with “shared circuits” may be important [11], these negative emotions might be experienced as unusual types of pain of more or less intensity.

As autism becomes more widely recognised, all doctors in every specialty will number individuals with ASD among their patients. Pain being such a common and important feature of pathophysiology, it will be important to be able to recognise pain as a possible cause of emotional distress and not to presume that pain is absent when its presentation is atypical. The use of an analogue pain scale, or of an informant-report scale [12, 13], may help such assessments.

In terms of recognising oversensitivity to pain in people with autism, it may be significant that some of the most widely used diagnostic instruments (e.g., [14–16]) do not specifically mention this as a feature (though they do include pain hyposensitivity). Indeed, a reason for the low reported incidence of pain hypersensitivity in autism is that it is not routinely sought after. The CARS-2 questionnaire [17] does consider indeed pain oversensitivity, but as an “overreaction,” and this may reflect a misunderstanding in the degree to which people with autism actually experience pain.

Some have suggested [18] that pain experts may fail to consider autism in the differential diagnosis of chronic and unusual pain, and our experience bears this out. A more widespread familiarity of all doctors dealing with children with the sensory and communications features of Autism Spectrum Disorders may lead to more prompt identification of ASD. Further research is required to identify the incidence of ASD in children with unexplained chronic pain. The development of specific pain assessment instruments for use with people with autism may be useful.

## 6. Conclusion

Autism is relatively common, with a prevalence of around 1% of the population. Affected individuals may experience sensations in all modalities with raised or lowered thresholds and sometimes in qualitatively abnormal ways. Amplified pain may be a presenting feature of autism, and its unusual character may delay diagnosis when individuals present to general physicians.

## Abbreviations

ASD: Autism Spectrum Disorder
H.S.E.: Health Service Executive.
